# Lignocellulosic fiber reinforcement in PPRC composites: An analysis of structural and thermal enhancements

**DOI:** 10.1371/journal.pone.0309128

**Published:** 2024-11-15

**Authors:** Fahad Ali Rabbani, Saima Yasin, Tanveer Iqbal, Hamayoun Mahmood, M. A. Mujtaba, Yasser Fouad, Manzoore Elahi M. Soudagar, M. A. Kalam

**Affiliations:** 1 Department of Chemical, Polymer, and Composite Materials Engineering, UET Lahore, Kala Shah Kaku, Pakistan; 2 Department of Chemical Engineering, UET Lahore, Lahore, Pakistan; 3 Department of Mechanical Engineering, UET Lahore, Kala Shah Kaku, Pakistan; 4 Department of Applied Mechanical Engineering, College of Applied Engineering, Muzahimiyah Branch, King Saud University, Riyadh, Saudi Arabia; 5 College of Engineering, Lishui University, Zhejiang, Lishui, China; 6 Department of Mechanical Engineering, Graphic Era (Deemed to be University), Dehradun, Uttarakhand, India; 7 School of Civil and Environmental Engineering, FEIT, University of Technology Sydney, Ultimo, NSW, Australia; Jazan University, SAUDI ARABIA

## Abstract

This study investigates the fabrication process of biocomposites and their resultant mechanical and thermal properties, essential for evaluating the performance of finished products. Polypropylene random copolymer (PPRC) was employed as the matrix phase, while rice husk (RH), a biowaste filler, was incorporated in varying concentrations. The rice husk fiber was treated with alkali (RHT) to enhance its lignocellulosic content. To improve interfacial bonding, maleic anhydride and NaOH treatment were utilized. Glass fiber grafted on polypropylene (PPGF) and talc powder functioned as additives. Both raw and treated rice husk fibers were characterized using Fourier-transform infrared spectroscopy (FTIR), field emission scanning electron microscopy (FESEM), and analytical methods to quantify the composition of lignin, cellulose, hemicellulose, and ash. Significant structural changes were observed, with cellulose content increasing from 26% to 53%. Wood polymer composites (WPC) produced from raw and treated rice husk were evaluated based on morphological studies, Izod impact testing, water absorption, heat distortion temperature (HDT), and VICAT softening temperature (VST). The results demonstrated that the HDT and VST of WPC improved by 24% and 7%, respectively, compared to PPRC, indicating enhanced structural and thermal properties. Additionally, impact strength and water absorption were found to be dependent on cellulose concentrations in the biocomposite. This study underscores the environmental benefits of utilizing biowaste rice husk in biocomposites, promoting sustainability by converting agricultural waste into valuable materials with enhanced properties for various industrial applications.

## 1. Introduction

Wood polymer biocomposites (WPCs) have emerged as the forefront in sustainable material science, offering a promising avenue to reduce our reliance on non-renewable resources and address pressing environmental concerns [1–3]. These composites represent a harmonious blend of organic and synthetic materials, bridging the gap between traditional wood products and modern polymers. A significant advancement in WPCs is the integration of biowaste, encompassing agricultural and forestry residues, as primary fillers [4–7]. These biowastes, such as rice husk, coconut coir, and bagasse, are abundant and renewable and present a cost-effective alternative to conventional fillers [8–12]. Rich in lignocellulosic fibers, they inherently enhance WPCs’ mechanical and thermal properties, making them more robust and versatile [13, 14].

This comprehensive review delves deep into the progressive landscape of WPCs, emphasizing the pivotal role of biowaste as the primary filler. It critically examines its profound influence on mechanical, thermal, and environmental attributes and explores its potential applications in various industries [15–18].

The strategic choice of fillers in WPCs is paramount in determining their overall performance and application range. While synthetic fillers like talc and glass microspheres have been extensively researched, biowaste-fillers have shown unparalleled promise in augmenting WPC characteristics. Numerous studies have underscored the multifaceted benefits of biowaste fillers, showcasing marked improvements in tensile strength, flexural strength, and impact resistance, among other mechanical properties [19–21]. For instance, Lemos et al. comprehensively analyzed the positive effects of high biowaste filler loading in a polypropylene matrix, emphasizing its transformative impact [17]. Such integrations bolster mechanical properties and champion environmental sustainability, positioning WPCs as a beacon for green material science.

However, the journey of integrating biowaste into WPCs is not devoid of challenges. One of the most formidable obstacles is achieving robust interfacial adhesion between the inherently hydrophilic nature of biowaste and the predominantly hydrophobic polymers. Surface treatments, such as Maleic Anhydride (MA), have emerged as game-changers in this domain. They enhance this adhesion by introducing functional groups that foster a stronger bond with the matrix, ensuring a seamless integration [22–24]. Another challenge is the moisture absorption propensity of biowaste, which can compromise the composite’s structural integrity and longevity. Innovative solutions, such as moisture barrier coatings or hydrophobic additives, have been developed to mitigate moisture uptake, ensuring the composite remains resilient even in humid environments [25–27]. Furthermore, ensuring a uniform biowaste dispersion within the polymer matrix is vital for achieving consistent and optimized properties. This is addressed through state-of-the-art mixing methods and meticulous preprocessing techniques [28].

Biodegradability, while an asset in eco-friendly contexts, may not always be desired, especially in applications demanding long-term durability. Incorporating biodegradation-resistant polymers, such as polypropylene (PP) or polyethylene (PE), can significantly enhance the composite’s overall durability and lifespan [29]. Processing challenges, especially those associated with the unique nature of biowaste fillers, can be adeptly tackled by adapting techniques and introducing modifiers to enhance workability [16, 30–33].

Wood polymer composites (WPC) are extensively used in various industries due to their superior mechanical properties, rigidity, and durability. They are ideal for construction applications such as railings, fences, docks, decks, roof tiles, and windows, as well as outdoor decking and landscaping, where high strength and resilience are essential [34, 35]. High heat distortion and softening temperatures make WPCs suitable for kitchen shelves and other moderate to high-temperature applications. The incorporation of biowaste into the polymer matrix reduces the cost of pure polymer products and enhances mechanical and thermal properties, offering a sustainable alternative to synthetic fillers [36]. This cost-effective approach promotes environmental sustainability. Additionally, WPCs are utilized in furniture manufacturing, automotive interior components, and a variety of consumer goods, including flooring, siding, and recreational equipment, highlighting their versatility and broad applicability [37, 38].

In our study, we embarked on a meticulous journey, utilizing rice husk as the biowaste filler and polypropylene random copolymer as the matrix to fabricate high-performance WPC sheets. Maleic anhydride was judiciously incorporated as a compatibilizer, and to further enhance mechanical and thermal properties, we integrated glass fiber grafted on polypropylene and talc. The materials underwent rigorous processing using a twin-screw reverse rotation mixer and were expertly molded with advanced thermal hydraulic compression techniques.

## 2. Raw materials

Compression-grade polypropylene random copolymer (melt index 0.25 g/10 min at 230°C, with constant load 2.16 kg and density 0.90 g/cm^3^), was purchased from Topiline, Seoul, Korea and used without pretreatment. Polypropylene reinforced with 30% glass fiber (PPGF, trade name KPG1030, and density 1.14 g/cm^3^) was purchased from Kopla Co., Ltd., Hwaseong, Korea. Maleic anhydride (MA) (98%) was purchased from Unichem, India. Biowaste rice husk (RH) was collected from local rice industries, Muridkay, Pakistan. NaOH (99%) was purchased from Sigma Aldrich, USA. Talc powder (TLC) was purchased from local supplier, Lahore, Pakistan.

## 3. Physio-chemical pretreatment

**Fig 1:**
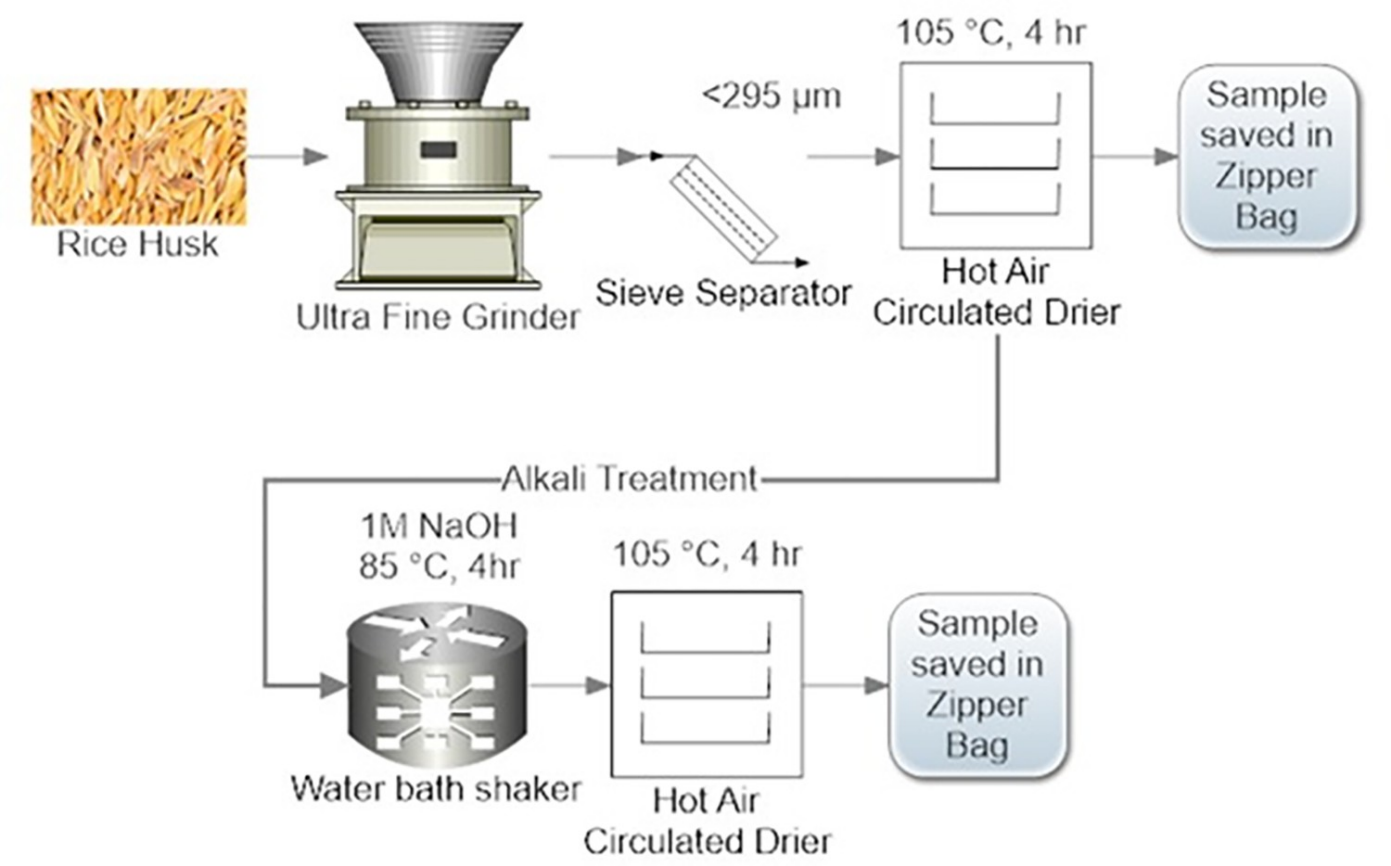
Biowaste rice husk pretreatment (physical top sequence, chemical bottom sequence).

Biowaste rice husk (RH) was cleaned, ground, and sieved to <295 μm size to achieve homogeneous mixing and uniform properties in the composite. Smaller particle sizes facilitate better dispersion and interfacial bonding, resulting in enhanced mechanical properties [39–41]. After drying, it underwent an alkali treatment using a method by Humayun et al., involving a 1 M NaOH solution and specific processing conditions [42]. The schematic procedure of physio-chemical pretreatment procedure of RH is presented in **Fig 1**, which outlines the collection, cleaning, sieving, drying, and alkali treatment steps [43].

## 4. Mixing of lignocellulosic biowaste rice husk with PPRC

**Fig 2:**
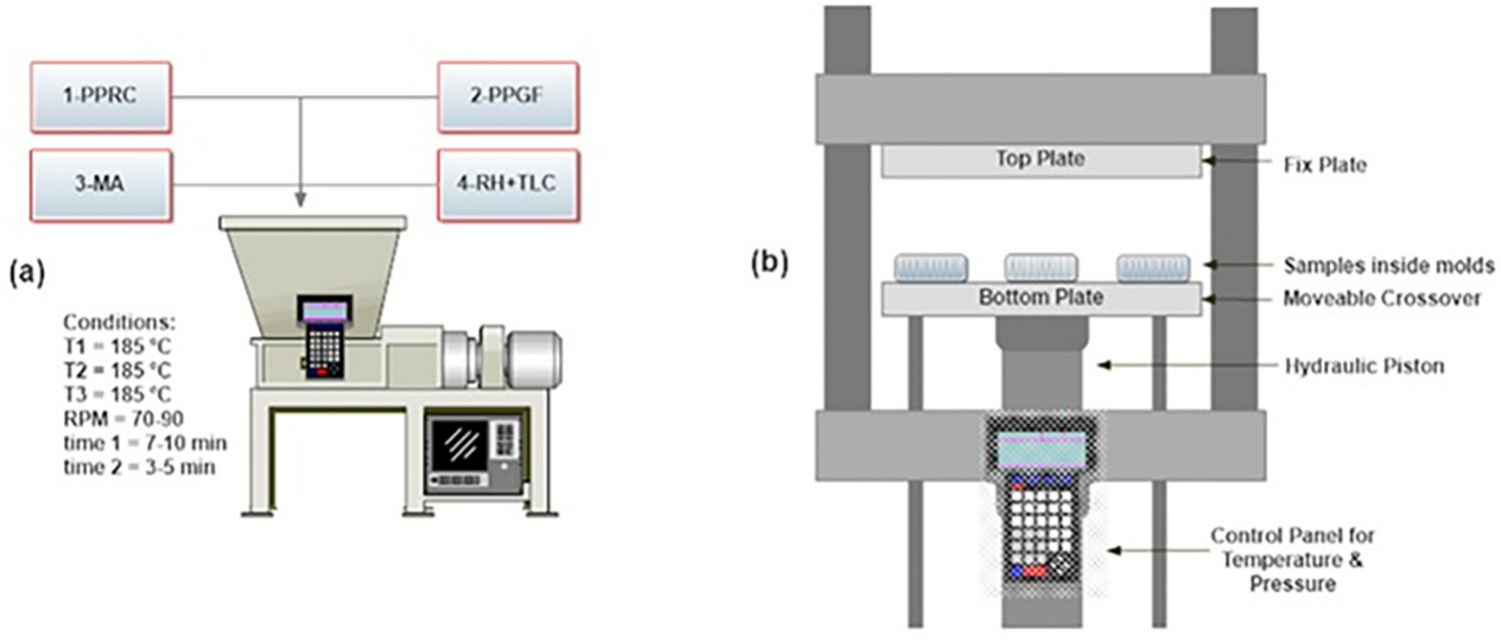
Fabrication of biocomposite (a) melt mixing, (b) thermal hydraulic compression molding.

Twin-screw batch internal mixer (Banbury internal mixer, model SBI-35L, Well Shyang Machinery Co., Ltd., Taiwan) was utilised to produce WPC as shown in **Fig 2A**. Three temperature zones and two helical mixers were included in the melt mixing chamber. The temperature of the heating zones was set between 180–190°C, slightly above the melting point of PPRC, to ensure adequate melting and account for heat losses [36]. The mixer speed (90 rpm) and mixing time (7–10 minutes) were optimized to ensure thorough dispersion of the rice husk within the PPRC matrix, achieving a homogeneous composite material while preserving the integrity of the components.

## 5. Fabrication of biocomposite

Following the completion of the compounding process, the semi-solid biocomposite was removed, placed into molds, and subjected to compression in a hydraulic thermal press (hydraulic platen press, Hartek Technologies Ltd., Guangzhou, China) for ten minutes as shown in **Fig 2B**. The temperature was maintained between 180–190°C, consistent with the mixing temperature, to ensure adequate melting and uniformity. A high pressure of 200 kPa was applied during compression to eliminate trapped air bubbles, resulting in a homogeneous, compact structure. After that, the heaters were turned off, and the samples were compressed until they reached room temperature while being cooled down. After the samples were prepared, they were taken out of the moulds and stored away for further analysis. The composition of the samples, expressed as a percentage, is presented in Table 1.

**Table 1:** Composition of raw materials in WPC panels.

Sample Name	RH	RHT	PPRC	PPGF	MA	TLC
PPRC	-	-	100	-	-	-
5RH	5	-	88	5	2	-
10RH	10	-	83	5	2	-
15RH	15	-	78	5	2	-
5RHT	-	5	88	5	2	-
10RHT	-	10	83	5	2	-
15RHT	-	15	78	5	2	-
5RH/2%TLC	5	-	86	5	2	2
10RH/2%TLC	10	-	81	5	2	2
15RH/2%TLC	15	-	76	5	2	2
5RHT/2%TLC	-	5	86	5	2	2
10RHT/2%TLC	-	10	81	5	2	2
15RHT/2%TLC	-	15	76	5	2	2

## 6. Characterization techniques

### 6.1 Fourier transform infrared spectroscopy (FTIR)

FTIR was used to identify structural changes that appeared due to alkali treatment of RH and WPC. JASCO FT/IR 4600 (Japan) was used to perform FTIR on different samples. The wavelength of spectrometer was set to 500–4000 cm^-1^ with scan resolution of 4 cm^-1^. Samples in the form of flat pallets were used to analyze functional groups appearance. The wavenumber input variable was observed as influencing the transmitted mode of radiation.

### 6.2 Lignocellulosic characterization

Lignocellulosic characterization of untreated and treated rice husk fibers was conducted according to standard analytical procedures. There are four main constitutes of rice husk fiber: cellulose, hemicellulose, lignin, and ash. Briefly, following procedures were followed:

## 6.2.1 Cellulose determination

In order to determine the amount of cellulose present in RH, the cellulose separation method was utilized. In a ratio of 10:1 (volume to volume), an acidic reagent that was composed of HNO_3_ and CH_3_COOH with an 80% w/w was created. Conical tubes containing 20 ml of liquid were filled with each of the samples, which weighed 0.1 g in total. Each tube received 3 mm of an acidic reagent that was stirred in. After thirty minutes in the boiling water, the test tubes were removed. After the tubes had been cooled, 10 ml of deionized water was added to each one. It was successful in removing impurities, and cellulose was found to have persisted in the mixture after it had been recovered with a diaphragm vacuum pump [44]. After drying the samples in open air for a period of 24 hours, the final step was to determine the cellulose content (in percent) of each sample.

## 6.2.2 Hemicellulose determination

The difference in holocellulose and cellulose content served as a basis for determining the presence of hemicellulose [45]. In a total volume of 30 mL of distilled water, 0.5 g of untreated or treated RH, 0.4 g of sodium chlorite (NaClO_2_), and 0.04 mL of acetic acid were added. The reaction mixture was put into a water bath and heated to 75 degrees Celsius while being stirred at a rate of 150 revolutions per minute. Following that, 0.2 g of NaClO_2_ and 0.04 mL of acetic acid were each added into the reaction mixture after an interval of 1 hour for a total of 2 hours of reaction time. Following the completion of the allotted time for the reaction, the mixture was filtered and washed between two and three times with distilled water in order to remove any remaining contaminants. The residue that was left on the filter paper was dried in an electric oven at a temperature of 80°C for twenty-four hours. After drying, the sample consisted primarily of holocellulose. The difference in holocellulose and cellulose content was used as a basis for determining the amount of hemicellulose present.

## 6.2.3 Ash determination

Ash content of RH treated and untreated was calculated by using muffle furnace [46]. A sample of known weight was burnt in excess air at the temperature 550°C for 2 hours unless all organic and volatile material are burnt out and only ash remained in crucible. Percentage ash was calculated using:

$$Ash\;yield\;(\%)=\frac{weight\;of\;ash}{amount\;of\;precombustion\;sample\;}x100$$

## 6.2.4 Lignin determination

Stoichiometric method was used to calculate lignin weight percent in RH sample by subtracting percentages of cellulose, hemicellulose, and ash from untreated sample. The stoichiometric calculation has been used by Mansoor A. et al., for calculation of cellulose from pineapple biomass [13].

### 6.3 Morphological study (FESEM)

Field emission scanning electron microscope (FESEM), Helious Nanolab G3 UC (FEI company, USA) was used to analyze surface morphology of selected samples. The samples were nonconductive, so they were coated with platinum by using AGAR High Resolution Coater (AGB7234, UK,) before loading the samples to FESEM. Using this technique, all samples were magnified up to 10000x, allowing for a detailed analysis of structural changes at the microscopic level.

### 6.4 Izod impact testing (unnotched)

The universal pendulum impact tester (Ray-Ran UK) was used to calculate the impact strength of unnotched WPC samples under ASTM-D4812. Rectangular samples of 90mm x 13mm x 4mm were used for testing. The Izod impact test measures the material’s resistance to impact from a swinging pendulum, providing an indication of the toughness and energy absorption capacity of the WPC.

### 6.5 Water absorption

Water absorption test for WPC sheets was performed against ASTM-D570 with repeated immersion technique. The samples were directly immersed in distilled water and their weight gain was observed during fixed time intervals for 60 days. The moisture uptake at any time point “t” as a result of moisture sorption was calculated by the following equation:

$$Moisture\;uptake=\frac{W_{t}-W_{o}}{W_{o}}x100$$

Where, W_t_ and W_o_ indicate the weight of humid composite sample at time *t* and the initial dry weight of materials prior to exposure to water immersion.

### 6.6 Heat distortion temperature & Vicat softening point

Heat distortion temperature (HDT) and Vicat softening point (VSP) was analyzed using Ray Ran Advanced HDT Vicat Softening Point Apparatus (Model: RR/HDV2, UK). HDT was performed using 3-point bending setup under ASTM-D648. The HDT, also known as heat deflection temperature, is the temperature at which a polymer or plastic sample deforms under a specified load. This property is crucial in product design, engineering, and the manufacture of thermoplastic components [47]. Stress of 1.8 MPa was applied on rectangular samples (100 mm x 14 mm x 4 mm) by using unique binary weight system with the span length of 64 mm for flat-wise adjustment and heating rate was set to 120°C/hr. A motion sensor attached to stress applied setup recorded the movement of bending. As the sample bent to 0.25 mm ± 0.01 mm then the system recorded HDT value in temperature unit, °C.

Vicat softening temperature (VST), or Vicat hardness, determines the softening point for materials with no definite melting point, such as plastics. It is measured as the temperature at which a specimen is penetrated to a depth of 1 mm by a flat-ended needle with a 1 mm^2^ circular or square cross-section. VST was performed on WPC synthesized specimen by following ASTM-D1525. A specific indenter nib of 1 mm^2^ was used to measure thermal creeping effect. A uniform temperature ramp of 120°C/hr was set and 10 N load was applied on specimens using binary weight system. A motion sensor attached to the indenter needle measured its movement and as the penetration reached to 1 mm ± 0.01 mm the result was recorded in Ray Ran apparatus in form of temperature°C.

## 7. Results and Discussion

### 7.1 Characterization of physio chemical pretreatment of lignocellulosic biowaste rice husk

#### 7.1.1 Chemical composition of rice husk

**Fig 3:**
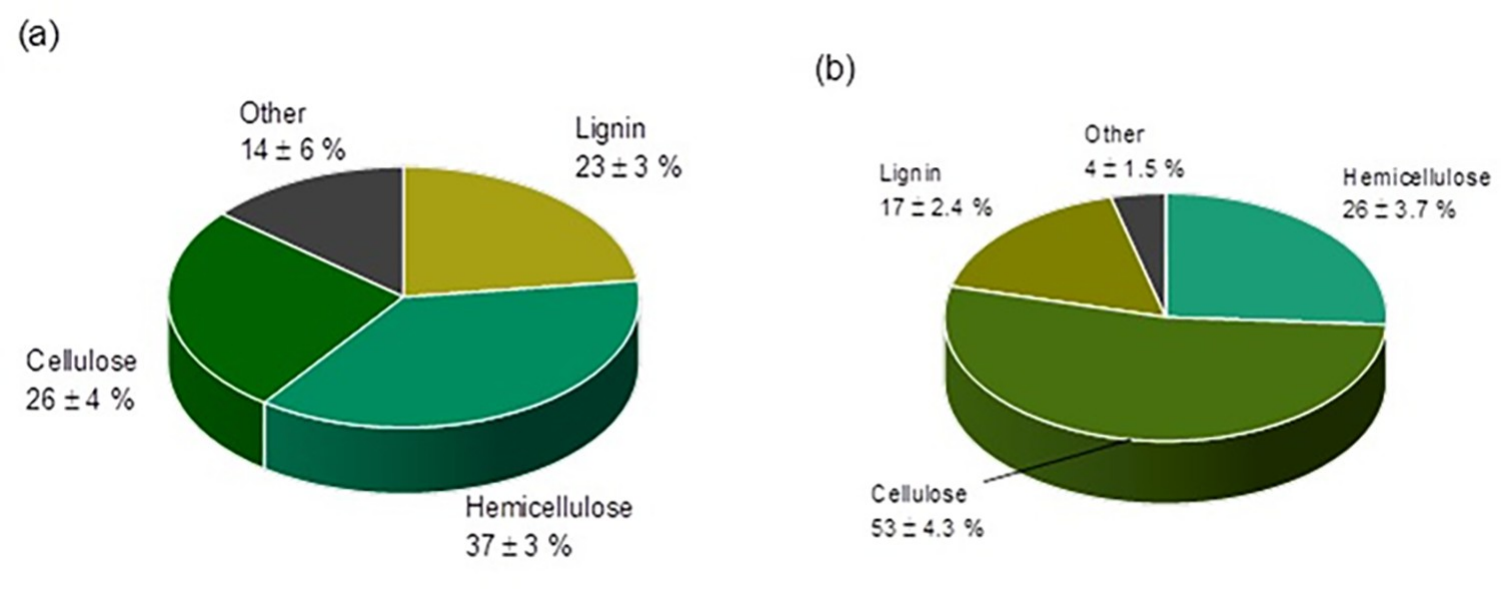
Composition of Rice Husk (a) Untreated, (b) Alkali Treated.

The lignocellulosic composition of untreated and alkali treated biowaste rice husk is shown in **Fig 3**. It was observed that untreated biowaste filler comprised of 26% cellulose, 37% hemicellulose, 23% lignin and 14% other components (minerals, ash, moisture). Whereas, after pretreatment with strong alkali solution the composition of fiber changed to 53%, 26%, 17%, and 4% of cellulose, hemicellulose, lignin, and other components, respectively. This data indicates that alkali treatment brought major changes in structure of RH fiber and cellulose rich fiber is obtained afterword. Pretreatment with NaOH marginally modified the color of redeveloped fiber from light brown to brown, indicating predicted modification (partial removal of hemicellulose and lignin, wax, fatty material, etc.). Cellulose has the highest specific strength and modulus in lignocellulosic fiber. To manufacture composites with lignocellulosic fiber as a reinforcement or filler in synthetic and biobased polymers, a high-cellulose fiber is required.

Semi-crystalline cellulose, amorphous hemicellulose, and lignin, which is a phenolpropanoid polymer, are the three primary components that make up lignocellulosic biomass [48]. Hemicellulose is the most abundant component of this type of biomass. It is made of carbohydrate component which is also present in cellulose [49]. The integrated impact that results from the complex chemistry and three-dimensional structure gives lignocellulosic biomass an exceptional degree of stability. Still there are some free protons present in cell wall that interacts with OH- groups present in alkali and therefore starts its decomposition. Bonds between lignocellulose and cellulose starts to break and swelling occurs [50]. Moving forward, alkali interacts with hydrogen and oxygen of hydroxyl group and change of electrons occurs from oxygen atoms-doners to hydrogen atoms-acceptors and dissolution of lignocellulose takes place [51]. As a consequence of this, some of the surface lignin and hemicellulose impurities become soluble, and the percentage portion of these impurities in the biomass that has been treated with alkali and regenerated is reduced [52].

Removal of amorphous hemicellulose and aromatic lignin causes increase in crystallinity of RH fiber due to pretreatment. Table 2 indicates the change in composition due to pretreatment. It was found that cellulose content increased in treated rice husk more than double that was present in untreated rice husk. Whereas concentrations of hemicellulose, lignin and other components decreased 30%, 26%, and 71% respectively. Hence pretreatment increased cellulosic content predominantly by removing other constitutes of rice husk fiber.

**Table 2:** Percent change in composition due to alkali treatment.

Rice Husk	Untreated (%)	Alkali treated (%)	Change (%)
Cellulose	26 ± 4	53 ± 4.3	+104
Hemicellulose	37 ± 3	26 ± 3.7	-30
Lignin	23 ± 3	17 ± 2.4	-26
Other	14 ± 6	4 ± 1.5	-71

The literature and the changes that occur in RH composition following an alkali pretreatment are in good agreement with one other. Mahmood H. et.al., studied effects of acid, alkali, hot-water, and ionic-liquid pretreatments on oil-palm fronds (OPF) fibers and indicated the change in composition of OPF fiber [42]. Their study indicated that highest recovery of cellulose was obtained in alkali process therefore alkali treatment process was opted and verified in this study as well. Lagerwall J. et.al., used acid hydrolysis of cellulose rich fibers to extract cellulosic nano crystals (CNC) and convert them into multifunctional thin films used as sensors [53]. Soleimani M. et al., studied flax fiber with alkali and bleach treatment ad found increase in cellulosic content as well [54]. Sulaiman M. et al., studied pretreatment of rice husk fiber with glycerol and choline and stated enrichment of cellulosic content in fiber used for packing applications [16]. Yu et al. (2014) [55] evaluated lignocellulosic corn stover after dilute acid and alkaline pretreatments. Acidic pretreatment removed 98% of hemicellulose and 5.4% of lignin, while alkali pretreatment removed 88.5% of original lignin and 47.3% of hemicellulose. Also, Camesasca et al., retreated napiergrass with dilute acidic and dilute basic solutions to improve biodegradability of lignocellulosic material [49]. They claimed that the combination of acidic and alkaline pretreatment was more efficient than acidic pretreatment on its own and improved the cellulose composition by as much as 58 percent while reducing the amount of hemicellulose elements to 10%.

These comparative results for different pretreatment techniques in terms of changes in the lignocellulosic composition of biomass made it abundantly clear that the pretreatment of lignocellulosic fiber with alkali was superior in terms of delignification of fiber as compared to that of acidic and hot water pretreatment. It is important to note, however, that a universal trend for different pretreatment technologies is extremely hard to extrapolate because of their complex interactions with lignocellulosic materials, which are in turn highly dependent on numerous factors such as cationic and anionic structures, viscosity, polarity, solid to liquid ratio, and the broad spectrum of pretreatment conditions [56].

Additionally, it is possible to draw the conclusion that biomass pretreatment with alkaline solvents is a viable, reusable, and environmentally benign way for efficiently utilizing biomass as a filler in biocomposites.

## 7.1.2 Effect of pretreatment on morphology of RH

There are three types of fillers that are added for biocomposite fabrication. The rice husk plays a major role for controlling mechanical and thermal properties whereas talc in powder form acts as a surface modifier to fill voids and cracks over biocomposite surface.

Rice husk was obtained in the form of flakes and after removal of unwanted particles (dust, derby etc) it was sent for crushing and sieving, following drying under air circulation conditions. The powder RH was then alkali treated and after washing it was dried under air circulated condition. Samples from both untreated and treated rice husk were stored in zipper bags and sent for SEM investigation. Talc powder was sent for SEM testing without any modification/ treatment.

Scanning electron microscope provides fundamental information about surface morphology and physical structure. It is expected that after alkali treatment, a few components of rice husk like lignin, hemicellulose, silica, wax etc., would have been removed. Therefore, structural change of rice husk can alter major properties of biocomposite.

### 7.2 FESEM of lignocellulosic biowaste before treatment and after treatment

**Fig 4:**
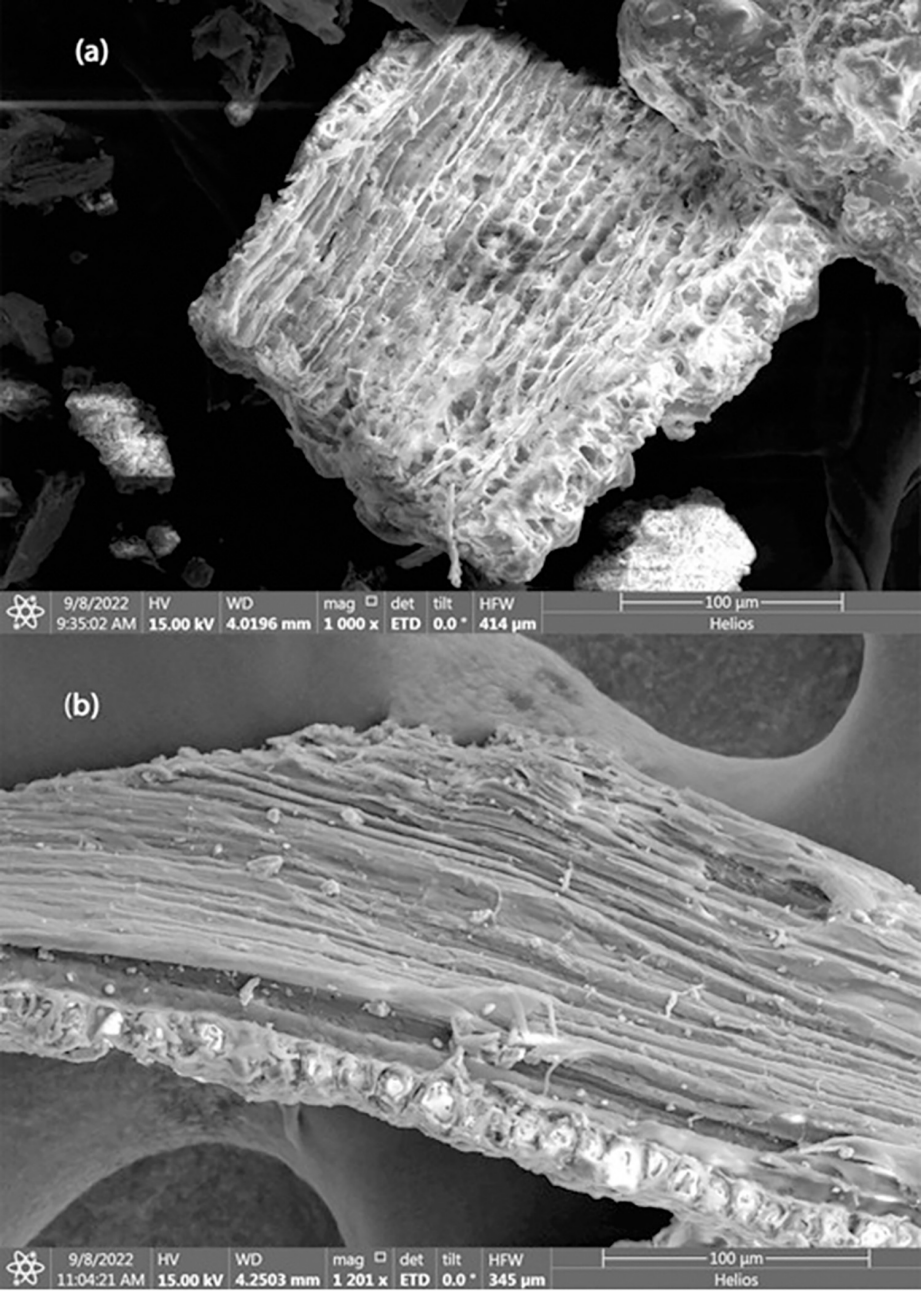
FESEM of (a)Untreated Rice Husk (b)Treated Rice Husk.

Particles size of less than 295 μm were collected after sieving and were sent for SEM characterization. **Fig 4A** represents surface morphology of untreated rice husk and **Fig 4B** represents alkali treated rice husk at magnification about 1000. It is clear from images that structural change occurred due to alkali treatment.

For untreated sample, RH flakes appeared to have cellulosic fiber bonded together with hemicellulose and lignin. Cross link web structure could be due to presence of lignin which binds cellulosic fiber and acts as a shell to save fiber bundles. Lignin also prevents biodegradation of rice husk fiber. There is a cloud like portions over cellulosic fiber bundle indicated presence of silica, wax and fats.

On the other hand, for treated RH, the structure of cellulosic bundles appears to be open, loose, and fiber length has shortened as shown in **Fig 4B**. Alkali treatment dissolves hemicellulose and lignin from RH fiber therefore cross-linkage has broken and spiked like fibral has appeared on surface. Structure of semi-crystalline/amorphous rice husk converted to crystalline structure. Further due to appearing of plain surface over cellulosic part, other impurities like wax, silica, and fats have washed out. Also, the material appears to be easily bending and twisting indicating soft tissues have prominent.

**Fig 5:**
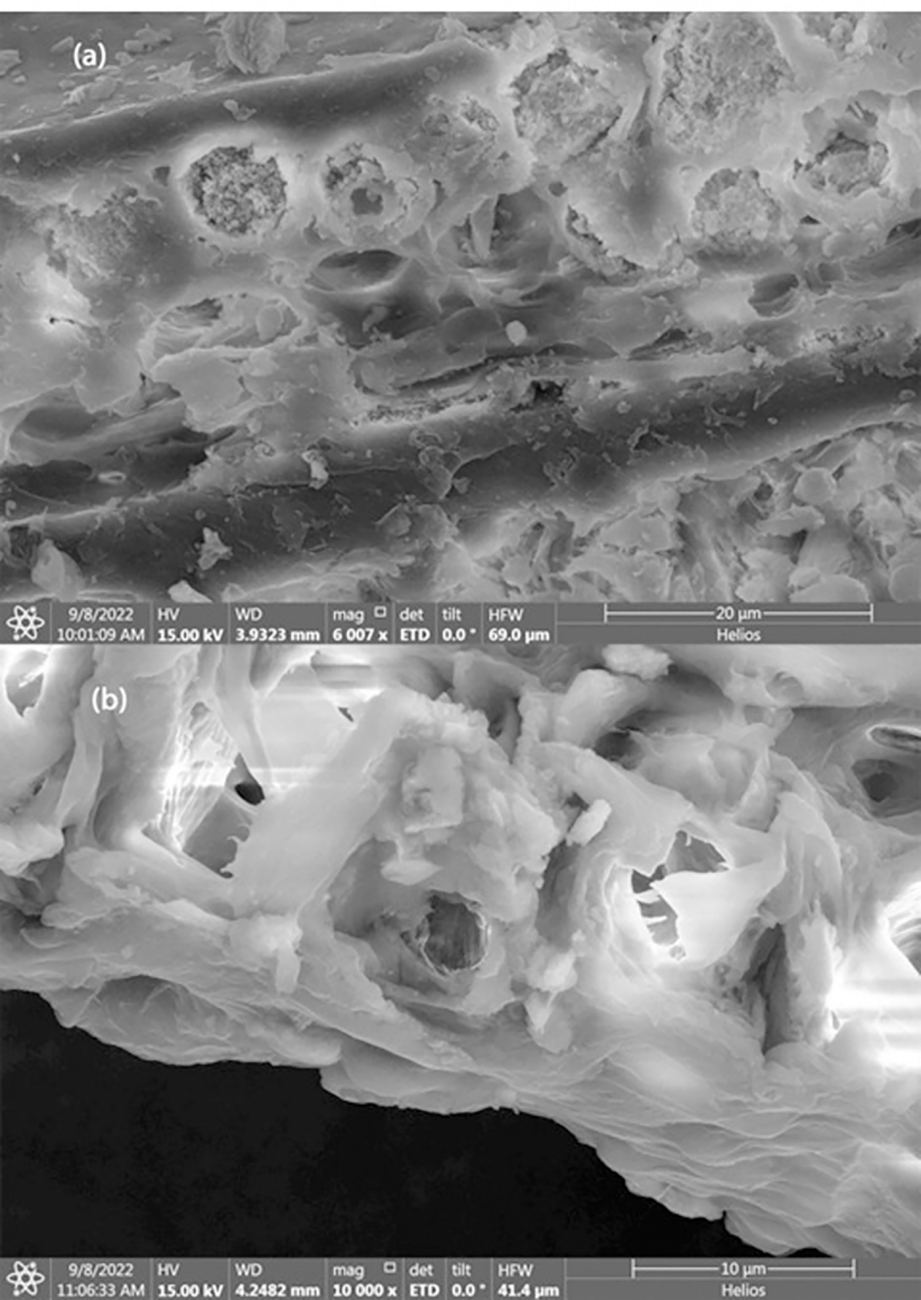
FESEM of Rice Husk at High Resolution (a) Untreated, (b) Alkali Treated.

Detailed FESEM micro images revealed alteration in fiber micro-pores due to alkali treatment. **Fig 5A** indicates cell wall of untreated RH fibers binded by hemicellulose-lignin matric. This matrix phase has many pits which have inorganic materials like silica and minerals. These minerals act like micro fillers inside rise husk fibers and increase its toughness and save them environmental effects (like moisture attack). After alkali treatment these pits were destructed due to removal of lignin from cell walls of fiber thus leading empty voids over fiber surface as shown in **Fig 5B**. Also, more active sites appear on cellulosic fibers due to the increase in surface area.

### 7.3 FESEM of inorganic filler-talc

**Fig 6:**
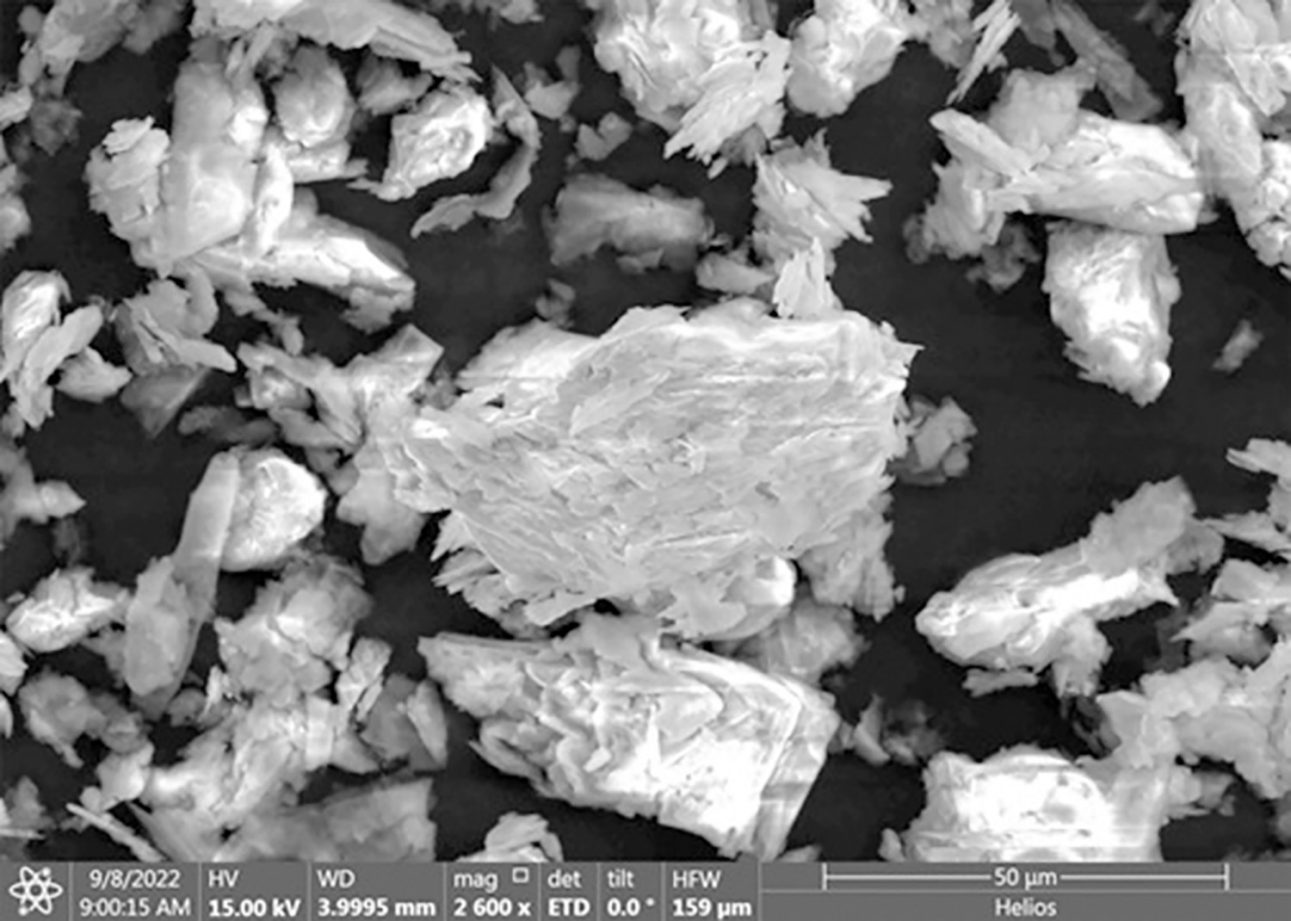
Talc used as a filler in WPC sheets.

FESEM micrographs of talc indicates that overall particle size range is 60–1 μm as indicated in **Fig 6**. Mostly particles are clusters of smaller ones making it lamella type structure. Some particles have spherical nest shape, but they are lesser in quantity. Close micrographs indicates that size of flat sheets of lamellae varies between 10–100 nm which are also discussed by Zazenski et al [57]. It also indicates that these sheets can slide over each other making them feasible to reshape under stress. Maleic anhydride compatibilizer help in dispersion of talc inorganic particles [58]. During extrusion process polymer-to-polymer chain forms bonding which effect melt flow index. In this process removal of polymeric materials become difficult thus, antilocking agents like silca or talc are added which help in easy removal of polymeric material from molds [59].

Talc is also added as an artificial physical foaming agent which helps to reduce/control density of composite materials [60]. Its lamella structure make it useful to act as a filler in contrast applications: ranging between filler to coating of materials [61].

## 7.3.1 FTIR of RH before and after chemical treatment

**Fig 7:**
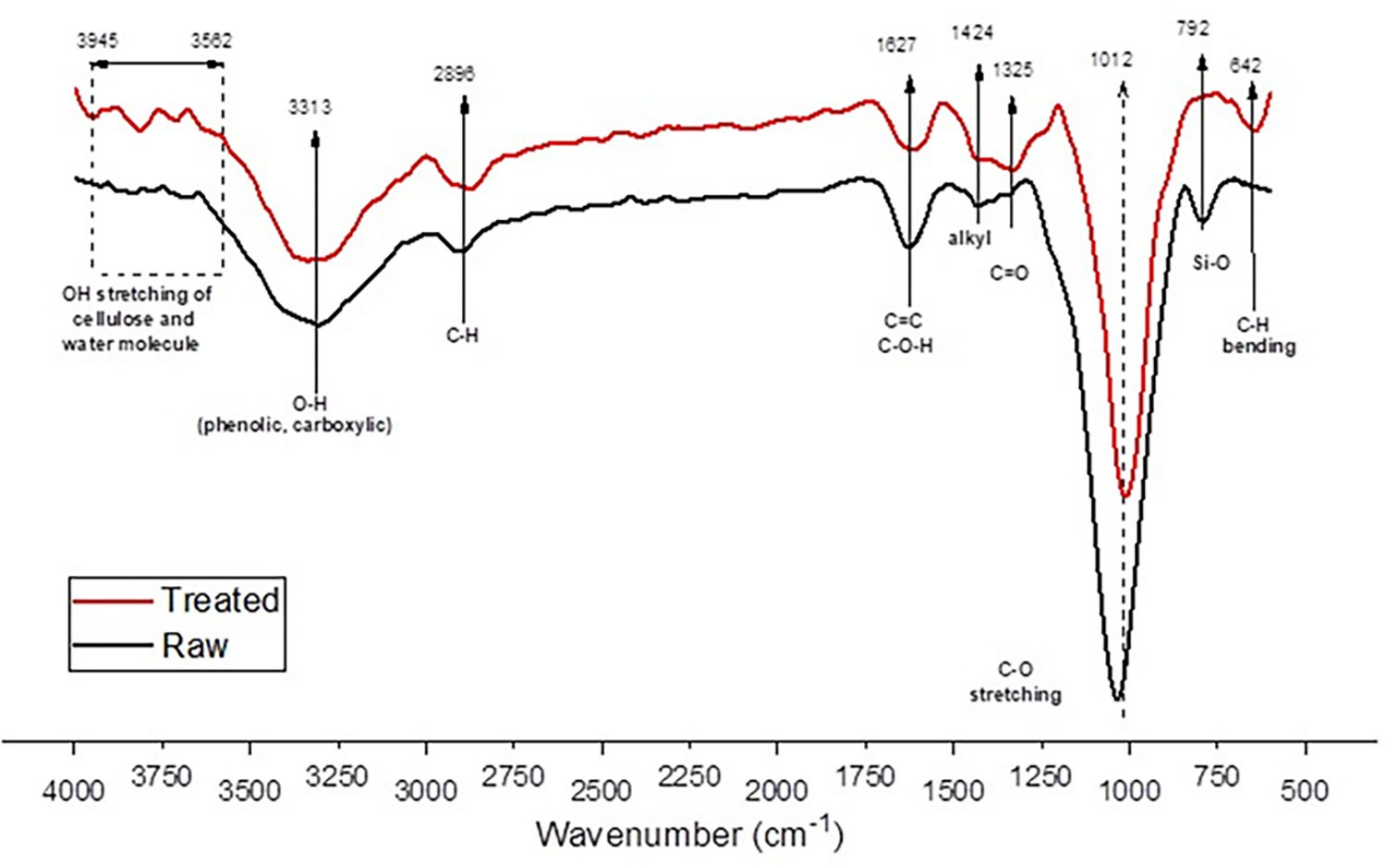
FTIR of untreated and treated rice husk.

Fourier transform infrared spectrometer (FTIR) was used over a wide range of wavenumber (500–4000) to identify change of surface-characteristics due to occurring of chemical functional groups of raw and surface modified rice husk as shown in **Fig 7**. It indicates the presence of different main peaks at 792, 1012, 1627, and 3313 cm^-1^, and stretching between 1325–1424 cm^-1^. These are indication of presence of silica, alkyl, alkene, carboxylic and alkene bonds.

After alkali treatment, C-H bending at 642 cm^-1^ and silica removal at 792 cm^-1^ were observed. Further the depth of C-O stretching at 1012 cm^-1^ decreased as compared to raw RH. This could be due to reduction of concentration of hemicellulose and lignin from the raw sample [62]. At the end of testing, vibration in peak-curve was observed between 3562–3945 cm^-1^ in treated sample which was not present in before in raw sample. This could be dye to formation of hydroxyl active sites on cellulosic part and also due to presence of water molecules [63]. Alkali treatment is used to create active -OH cites on surface of rice husk. These hydroxyl groups help in creating coupling with polymeric phase in presence of suitable compatibilizer. But this also results in an increase in hydrophilicity of treated cellulosic fiber. This result indicates that alkali pretreatment of RH did not cause any major structural change in sample. Further, cellulosic part did not destroy, and silica was also removed from raw RH [64].

Further the spectrum with a high absorption of band at approximately 3299 cm^-1^ for untreated rice husk. This band corresponds to cellulosic fiber which stretches due to vibration of hydroxyl (-OH) groups [65]. This band shifts to 3313 cm^-1^ for alkali treated rice husk indicating presence of free -OH groups. These free functional groups appear in carboxylic and phenolic groups during treatment process, and they do not take part in hydrogen bonding [66]. Also, there is a decline in intensity in this region which could be due to chemical reaction of cellulosic -OH group with alkali NaOH. Alkyl (CH) group present was observed at band width 2896 cm^-1^ which is associated with saturated hydrocarbon chain present in cellulose structure [67].

Intensity for treated sample increased which indicates concentration of cellulosic component increased after treatment. Another absorption vibration was observed at 1627 cm^-1^ for raw rice husk sample indicates presence of wax and natural fats with carboxylic functional groups in easter linkages. Alkali treatment removed wax and natural fats, thus the intensity of vibration peaks decreased. Peaks of 1012 cm^-1^ and 1015 cm^-1^ could be designated to COC group in glucose structure of cellulose [68]. Silica stretching vibration appeared at 792 cm^-1^ for untreated sample in O-Si-O group. This vibration does not appear for treated sample, therefore evident removal from surface modification. At final stage of peak evaluation, a stretching vibration at 642 cm^-1^ appeared in treated rice husk which was not present in raw rice husk. This peak corresponds to alkyl bending on cellulosic side which helps in formation of new bonds.

## 8. WPC panels characterizations

### 8.1 Morphological study-FESEM

**Fig 8:**
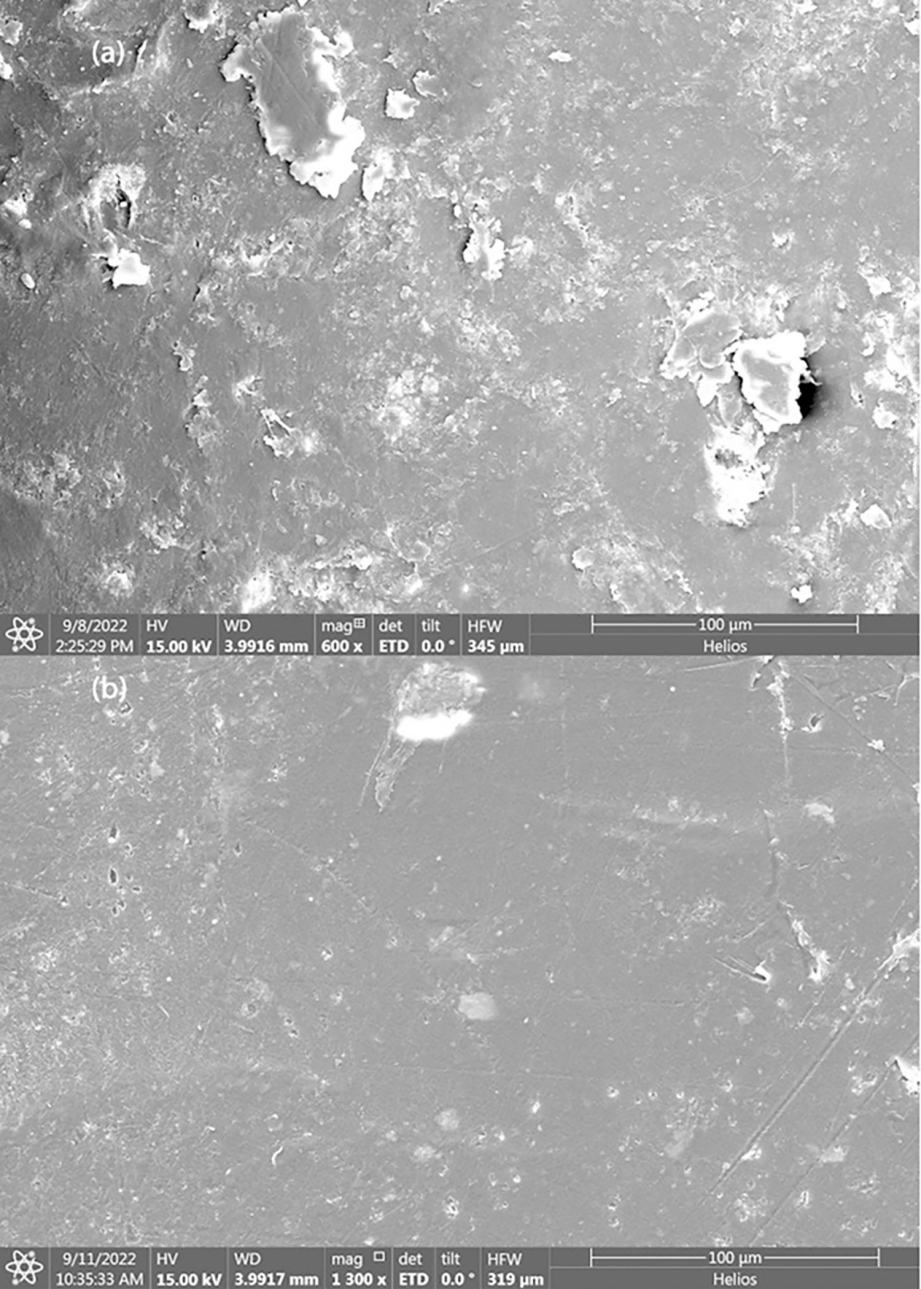
FESEM of Biocomposite Manufactured by (a) Untreated Rice Husk,(b) Treated Rice Husk.

**Fig 8A and 8B** reveal the distinct morphological differences between the biocomposites made from untreated RH and RHT. The biocomposite with untreated RH exhibits a rough surface with numerous surface defects, indicating a lack of proper interfacial adhesion between the fibers and the polymer matrix. On the other hand, the biocomposite with treated RHT demonstrates a smoother surface with fewer defects, implying enhanced interfacial adhesion and better bonding between the fibers and the polymer matrix.

The observed differences in morphology and surface characteristics are attributed to the effect of aspect ratio and fiber treatment [69]. Untreated RH possesses a higher aspect ratio, which might hinder proper adhesion and stress transfer within the composite, leading to a rough surface with more defects. In contrast, the lower aspect ratio of alkali treated RHT promotes better compatibility with the polymer matrix, resulting in a smoother surface and improved interfacial bonding.

This parallel comparison emphasizes the significance of fiber treatment and aspect ratio in influencing the surface characteristics and adhesion within the wood polymer composite. Proper fiber treatment and optimization of aspect ratio can lead to enhanced bonding between the fibers and the polymer matrix, thereby positively affecting the mechanical properties of the biocomposite.

### 8.2 Izod impact properties

**Fig 9:**
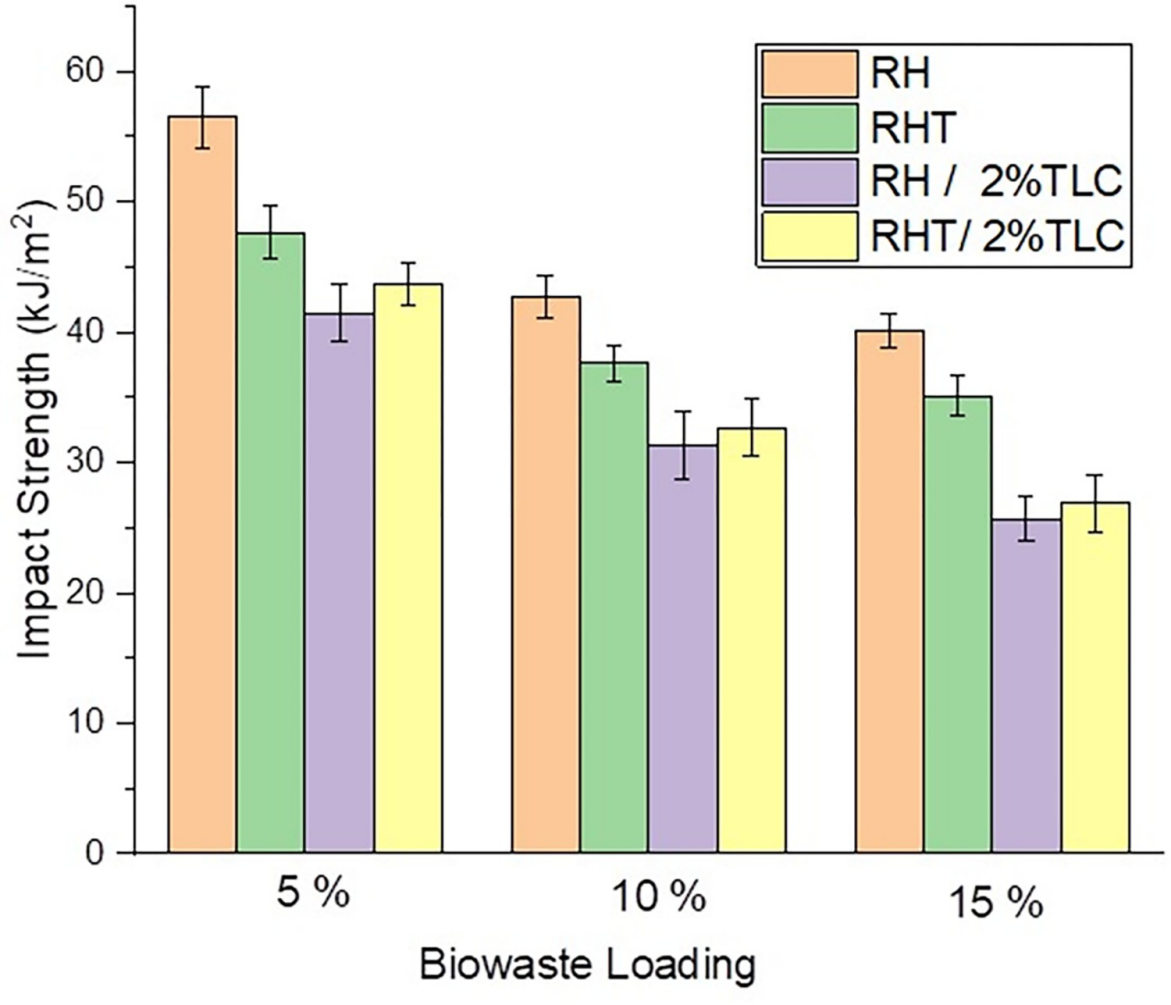
Average impact strength of WPC sheets ± standard deviation included.

The Izod unnotched impact test was performed on various wood polymer composite (WPC) samples, and the results are presented in **Fig 9**. Neat polypropylene (PPRC) did not show measurable impact strength as it remained unbroken even under high hammer impact and demonstrated plastic deformation, consistent with previous reports by Mijiyawa et al. [70]. The high intermolecular forces and hydrogen bonding between polymeric chains in neat PPRC contribute to its flexibility and impact resistance.

The impact strength for untreated RH-based WPC was 56±2 kJ/m^2^ for 5% loading, and it decreased to 42 kJ/m^2^ and 40 kJ/m^2^ for 10% and 15% loading, respectively. This represents a 24% decrease in strength with a 5% to 10% increase in RH loading and a 6% decrease with a 10% to 15% increase in RH loading. For WPC with alkali-treated RH, the impact strength ranged from 47 kJ/m^2^ to 35 kJ/m^2^, and there was a percent decrease of 21% and 5% for increments between 5% to 10% and 10% to 15%, respectively.

When 2% talc was incorporated as an inorganic filler in the WPC sheets, the overall filler content (organic + inorganic) increased, leading to a decrease in compatibility effects and resulting in the minimum impact strength for all fixed biowaste loadings. The impact strength for WPC samples with RH/2%TLC (talc) varied between 41–25 kJ/m^2^, with percent decreases of 24% and 19% for biowaste loading increases from 5% to 10% and 10% to 15%, respectively.

Comparing WPC samples with RH/2%TLC and RHT/2%TLC, it was observed that the impact strength for RHT/2%TLC was higher. SEM analysis micrographs revealed that treating RH with alkali washed out impurities, creating free pits and increasing the surface area of RH. This allowed talc particles to settle into these voids, leading to a better interlocking of fiber-matrix, thereby elevating the impact strength of the WPC. The impact strength for RHT/2%TLC ranged from 43–26 kJ/m^2^, with percent decreases of 25% and 18% for biowaste loading increases from 5% to 10% and 10% to 15%, respectively.

When examining fixed biowaste loading in the WPC, the overall behavior was repetitive. The maximum impact strength for 5% RH-WPC was 56 kJ/m^2^, while the minimum for 5% RHT/2%TLC WPC was 41 kJ/m^2^, representing a 26% decrease compared to the former. Overall trend of impact strength of WPC made from different fillers is RH>RHT>RHT/2%TLC>RH/2%TLC.

The aspect ratio of the fibers, affected by the alkali treatment, plays a role in the impact strength of WPC. Alkali treatment can reduce the fiber size or aspect ratio, which might contribute to the observed decrease in impact strength for WPC samples with treated RH [71]. On the other hand, untreated RH has a uniform aspect ratio, and the decrease in impact strength in untreated RH-based WPC is primarily attributed to the higher concentration of filler. It is important to note that filler loading, including both organic and inorganic fillers, still plays a significant role in optimizing the impact strength of WPC. The overall trend suggests that untreated RH samples exhibit the highest impact strength, while samples with an additional 2% talc show the lowest impact strength. Talc acts as a filler for micro-pores between the matrix and the fiber, but it also creates sliding of talc lamellae, as observed in the SEM micrographs, which leads to a decrease in impact mechanical strength.

**Fig 10:**
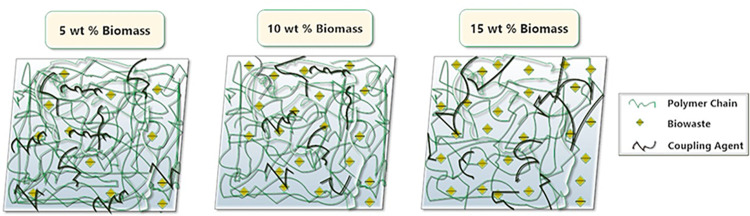
Intermolecular interaction of polymer chains with biowaste fibers and coupling agent.

Incorporating lignocellulosic fiber into PPRC, along with a coupling agent, resulted in a decrease in impact strength. This trend can be described by two phenomena. Firstly, polymer matrix chains become poorly bonded as a result of the inclusion of filler between the chain strands. So as the concentration of filler will increase, therefore, impact strength of the WPC will decrease. **Fig 10** illustrates this behavior. Secondly, impact strength is a function of aspect ratio of fiber. So, when RH fibers were treated with strong alkali solution, then fiber length decreased so the aspect ratio. Therefore, when RHT fibers were incorporated to fabricate WPC, then the impact strength showed lower value as compared to the corresponding concentration of untreated rice husk.

### 8.3 Heat distortion temperature /heat deflection temperature (HDT)

The heat distortion temperature (HDT) is an essential parameter for assessing the load-bearing capability of materials at elevated temperatures, making it crucial for biocomposite applications subjected to varying thermal conditions. As the temperature increases, the kinetic energy of polymeric chains rises, leading to a weakening of intermolecular forces in the material. HDT values provide crucial information for product design and material selection in commercial and industrial applications.

**Fig 11:**
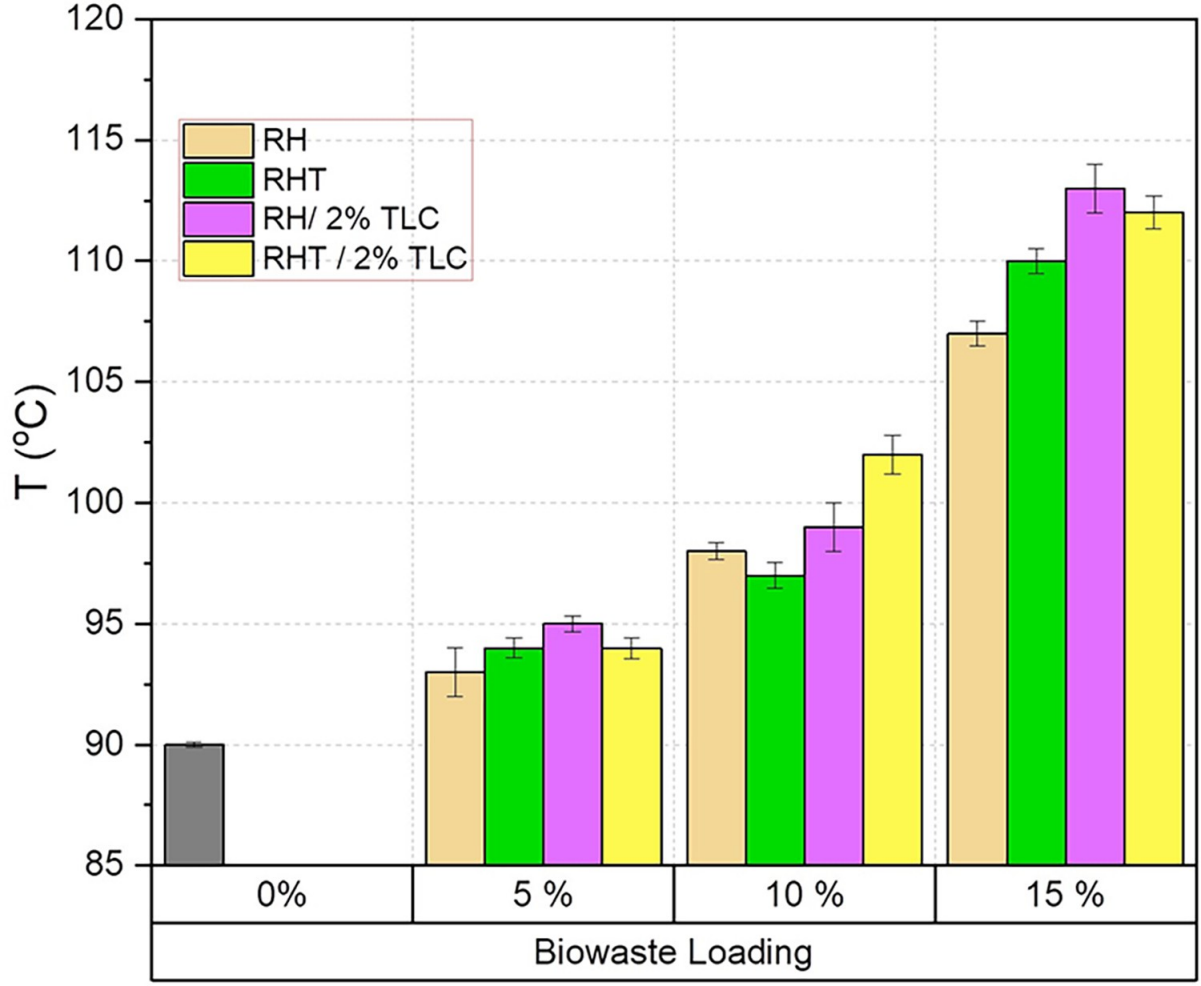
Heat deflection temperature of WPC fabricated by various filler ratio.

**Fig 11** illustrates the HDT values of neat PPRC and various wood polymer composites (WPC) as a function of different filler loadings. It is evident that HDT values increase with the increase in filler content.

The HDT of neat PPRC is 90°C, and it shows a slight enhancement when 5% of the filler (wood) is added to the polymer matrix. The HDT values for RH, RHT, RH/2%TLC, and RHT/2%TLC composites increased by approximately 3.33%, 4.44%, 5.55%, and 4.44%, respectively. For WPC sheets with a 10% biowaste loading, the maximum HDT value of 102°C was observed in RHT/2%TLC samples, which is approximately 13% higher than that of neat PPRC. Similarly, other WPC samples (RH, RHT, and RHT/2%TLC) showed HDT improvements of about 9%, 7%, and 10%, respectively, with 10% fiber loading. The addition of fillers to the polymer matrix slows down the mobility of polymer chains, resulting in improved stress-bearing capacity at elevated temperatures. WPC sheets with 15% biowaste loading demonstrated considerable HDT improvements compared to neat PPRC. Specifically, RH samples showed a 19% improvement, RHT samples showed a 22% improvement, RH/2%TLC samples showed a 26% improvement, and RHT/2%TLC samples showed a 24% improvement in HDT compared to their respective composites.

It is evident that the addition of inorganic filler (talc) contributed to the enhancement of HDT in all composites. Furthermore, the interaction between the polymer and filler particles, rather than the interaction between polymer molecules, played a significant role in improving HDT. This increased particle-polymer interaction was more pronounced in composites containing 17% filler.

The heat distortion temperature of polymers is influenced by various factors, including crystallinity and the presence of reinforcement agents or fillers. Previous studies have shown that reinforcing polymers with natural fibers can significantly increase the HDT value [72]. Additionally, smaller particles have a higher immobilization ability on polymer chains, leading to better HDT values in composites [73].

### 8.4 Thermal creep temperature / Vicat softening temperature (VST)

**Fig 12:**
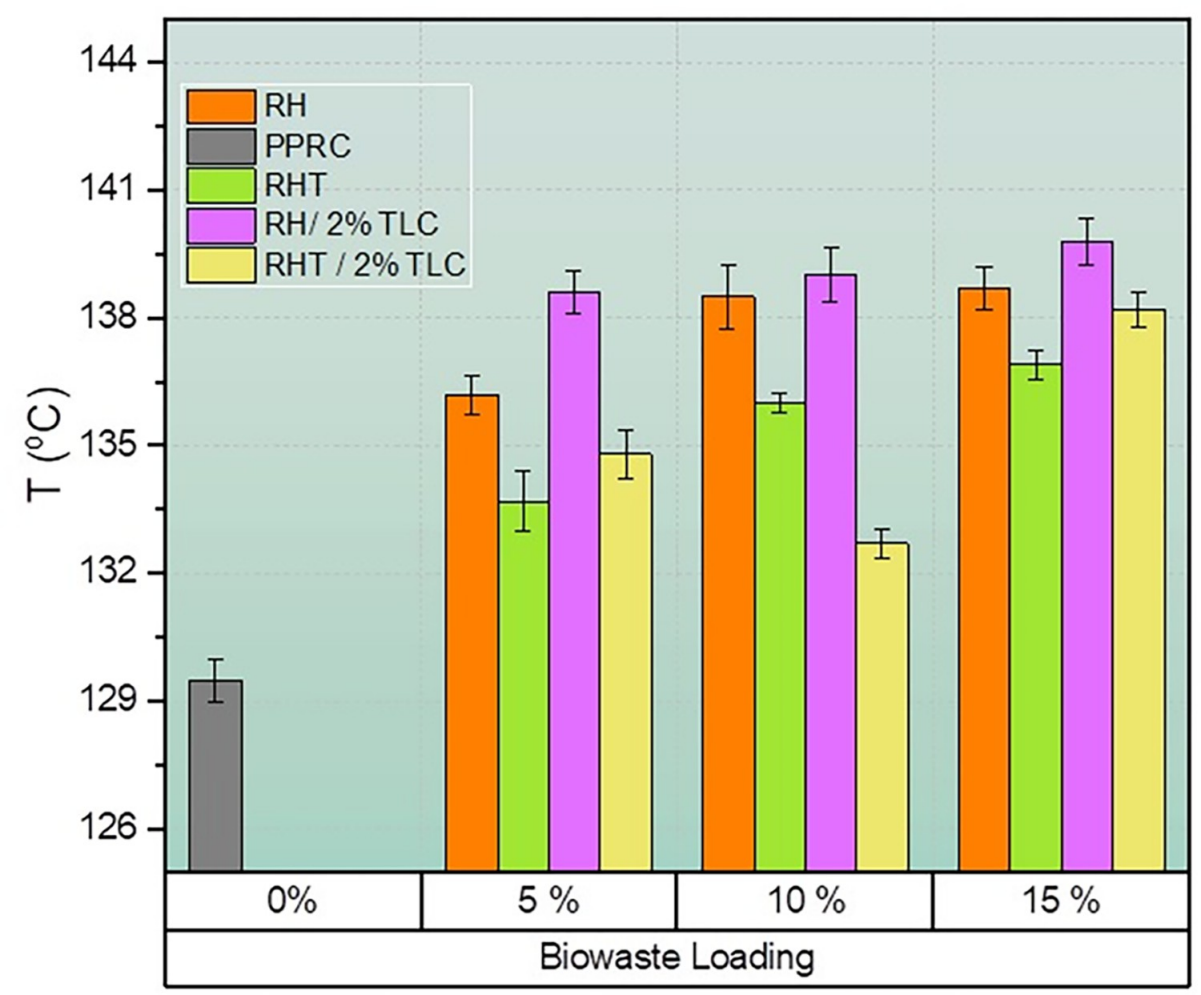
Vicat softening point temperature of WPC fabricated by various filler ratio.

**Fig 12** presents the thermal creep temperature (VST) values of neat PPRC and various wood polymer composites (WPC) as a function of different filler loadings. It is evident that the incorporation of biowaste fillers in neat PPRC matrix led to an improvement in VST values of the resulting WPC sheets, with an overall increase of 3–10°C.

Neat PPRC demonstrated a VST value of 129.5°C. The addition of raw rice husk in WPC resulted in approximately 5%, 7%, and 7% higher VST values for 5%, 10%, and 15% biowaste loading, respectively. Similarly, the WPC made from treated rice husk (RHT) showed an improvement in VST values compared to neat PPRC, with about 3%, 5%, and 6% increases observed for 5%, 10%, and 15% biowaste loading, respectively. This enhancement in VST could be attributed to the higher proportion of lignocellulosic content in the biowaste.

The incorporation of 2% talc in the biocomposite significantly increased the VST values. This can be attributed to the nanoscale talc particles filling the interfacial gaps between the biowaste fibers and the polymer matrix, reducing the mobility of molecular chains at elevated temperatures. This phenomenon was more pronounced in composites containing untreated rice husk, which had a denser and stiffer structure. Consequently, the highest VST value of 139.8°C was observed in WPC sheets with 15% untreated rice husk and 2% talc, representing an 8% improvement compared to neat PPRC. A similar increase of around 7% was observed for both 5% and 10% biowaste loading. For talc incorporation in treated rice husk-based composites, VST values improved by about 4%, 2%, and 7% for biowaste loadings of 5%, 10%, and 15%, respectively. The overall trend of VST values for all WPC sheets was RH/2%TLC >RH >RHT/2%TLC >RHT.

These results align with previous studies on natural fiber composites incorporating inorganic fillers. Wang et al. observed improved VST values for composites made from PVC and inorganic fillers [74]. Tasdemir et al. studied PP composites with cotton fibers and reported that a 3% cotton fiber loading increased VST, while a 6% loading decreased it [75]. However, some studies, including those by Roy et al. (2011), de Lemos et al. (2017), Kailasanathan et al. (2022), and Fambri et al., indicated that the improvements in VST values were relatively insignificant due to the homogeneity or phase continuity in the composites [17, 76–78]. In can be concluded that the addition of biowaste fillers, especially talc, in the WPC matrix resulted in enhanced thermal creep resistance as reflected in the improved VST values. The nanoscale reinforcement of talc in the composites played a crucial role in increasing the VST, making WPC sheets more suitable for applications requiring better temperature stability. These findings emphasize the potential of WPCs for various engineering and industrial applications where thermal performance is a critical factor in material selection.

### 8.5 Water absorption and thickness swelling

Lignocellulosic materials, such as wood, are hygroscopic, leading to weight increase and material swelling when exposed to humidity or direct water contact. This restricts their use to dry environments. However, Wood-Plastic Composites (WPCs), composed of lignocellulosic fibers, can have their water absorption and swelling controlled by adjusting the Lignocellulosic Fiber (LF) percentage, making them suitable for humid and wet environments[36, 79].

Polypropylene Random Copolymer (PPRC), a hydrophobic polymer, does not absorb water, making it ideal for water sanitary piping. The water absorption in biocomposites is primarily determined by the filler content, which forms voids and cracks on the WPC surface. As the filler content increases, so does the water absorption and swelling.

**Fig 13:**
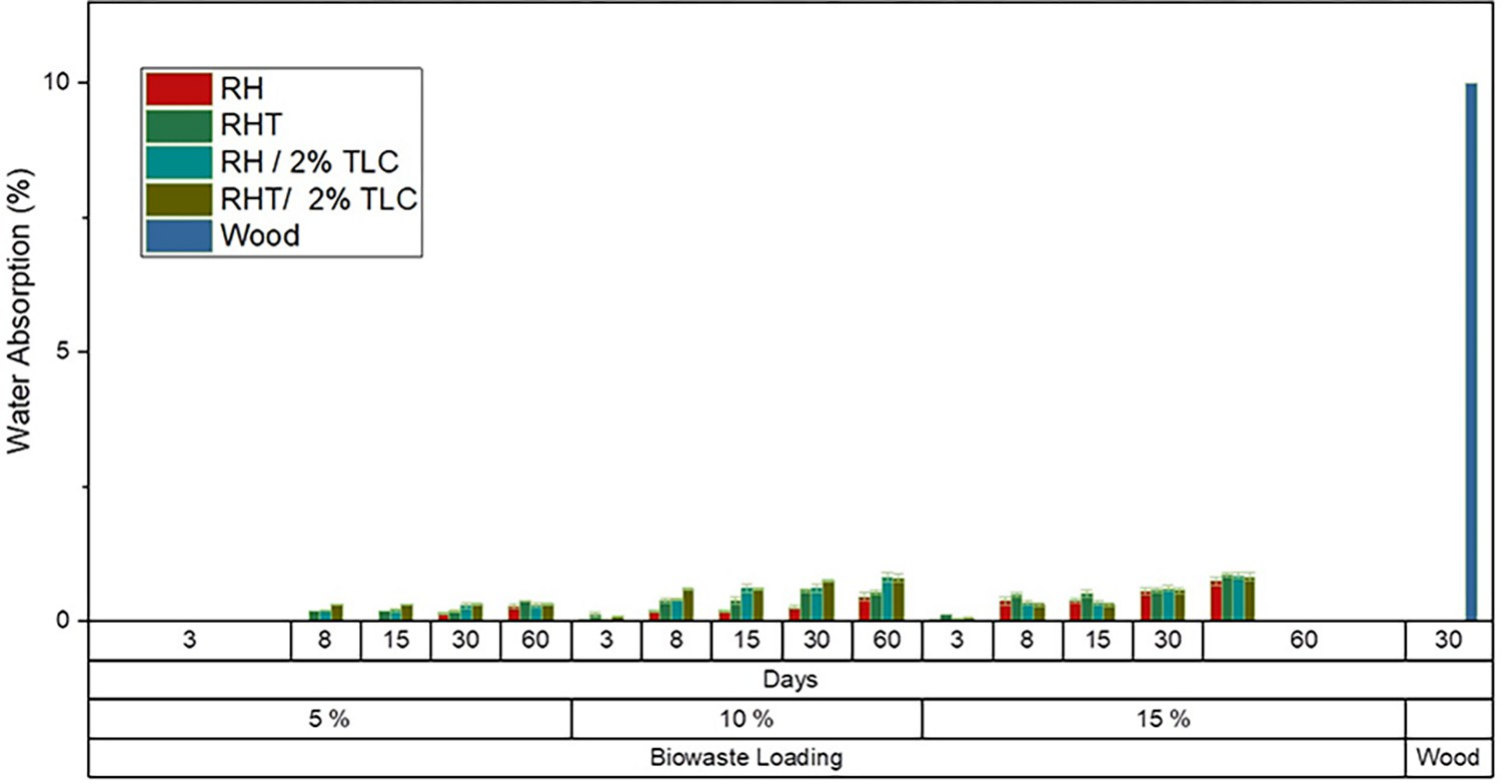
Water absorption timeline of WPC fabricated by various filler ratio for 60 days.

**Fig 13** shows the water absorption of WPC samples immersed in water. After three days, no water absorption was recorded for 5% BW loading, while a maximum weight increase of 0.04% and 0.14% was observed for 10% and 15% BW loading, respectively. Over a 60-day period, the maximum water absorption was 0.36% for 5% biowaste loading, 0.8% for 10% loading, and 0.84% for 15% loading. Despite this, no visible or measurable thickness swelling was observed after 60 days of immersion.

Anderson et al. proposed a three-step absorption behavior when wood is directly immersed in water. Initially, water diffuses into the pores, resulting in a rapid weight gain. As water contacts organic or soluble materials in the cell walls of the wood fiber, further water uptake occurs, albeit at a slower rate. In the final stage, water interacts with the hemicellulose and cellulose components of the wood fiber, causing swelling [80]. In comparison, the synthesized WPC sheet only exhibits the first phase of this process, with no observable swelling after 60 days of direct immersion in water.

## 9. Future work and recommendations

Future research should incorporate advanced analytical techniques, such as molecular dynamics simulations, to provide a deeper understanding of the interactions between biowaste fillers and the polymer matrix. Long-term durability studies under varying environmental conditions are essential to assess the real-world applicability and lifespan of these composites. Additionally, investigating the use of hydrophobic additives and surface modifications could further reduce water absorption in high-filler-content composites. Emphasizing the environmental benefits and practical applications of these materials, such as in outdoor construction, automotive components, and consumer goods, will align future work with current trends towards greener and more sustainable technologies.

## 10. Conclusions

This study utilized polypropylene random copolymer (PPRC) to investigate the interaction of various organic and inorganic fillers. Biowaste rice husk was treated to extract cellulose via alkali treatment method. The influence of alkali treatment created cellulose rich biowaste fiber and morphology modified as evident by FESEM and FTIR. Cellulose, hemicellulose, lignin, and other components (ash, moisture, fats, volatile) increased from 26% ±4, 37% ±3, 23%±3, and 14% ±6 for the untreated sample to 53% ±4.3, 26% ±3.7, 17% ±2.4, and 4% ±1.5 for the treated sample respectively. Biocomposite panels made from different compositions of raw materials possessed a range of modification in their structural, mechanical, and thermal properties. Overall trend of impact strength of WPC panels made from different bio fillers was RH>RHT>RHT/2%TLC>RH/2%TLC. According to the results of the thermal stability test, the heat deflection temperature and the thermal creep temperature of WPC panels are both improved by 26% and 15% over those of pure PPRC, respectively. Overall thermal creep temperature value trend for all WPC sheets was: RHT<RHT/2%TLC<RH<RH/2%TLC. According to the findings, the amount of water absorbed by any composition of WPC panels during a long-term immersion of samples in water for a period of sixty days was less than one percent.

## Supporting information

S1 File (XLSX)
